# Colorectal Cancer Invasion and Atrophy of the Enteric Nervous System: Potential Feedback and Impact on Cancer Progression

**DOI:** 10.3390/ijms21093391

**Published:** 2020-05-11

**Authors:** Janusz Godlewski, Zbigniew Kmiec

**Affiliations:** 1 Department of Human Histology and Embryology, Collegium Medicum, School of Medicine, University of Warmia and Mazury, 10-082 Olsztyn, Poland; 2 Department of Histology, Medical University of Gdansk, 80-210 Gdansk, Poland; zkmiec@gumed.edu.pl

**Keywords:** colorectal cancer, cancer invasion, tumour microenvironment, enteric nervous system, neuropeptides, galanin, Ach, perineural invasion, neurotrophins, TrkB

## Abstract

Colorectal cancer (CRC) invasion within the large intestine wall results in the replacement of normal tissue architecture by tumour mass. Cancer cells digest the extracellular matrix (ECM) by the release of proteolytic enzymes. The disintegration of matrix ground substance activates several deposited growth factors which stimulate cell proliferation. Stromal (mainly fibroblasts), immune and cancer cells dominate in this area and become involved in a network of multimodal interactions which significantly induce proliferation of colon cancer cells, inhibit their apoptosis and promote their spreading within the local tumour microenvironment. Cancer invasion destroys nerve fibres and neurons of the local enteric nervous system (ENS) and induces subsequent atrophy of the submucosal and myenteric plexuses in areas adjacent to the cancer boundary. Interestingly, the reduction of plexuses’ size is accompanied by the increased number of galanin-immunoreactive neurons and increased galanin content in parts of the colon located close to the tumour. Galanin, a neuroprotective peptide, may inhibit the extrinsic pathway of apoptosis and in this way promote cancer cell survival. The possible role of acetylcholine and some ENS neuropeptides was also discussed. Invasion of cancer cells spreads along nerve fibres with the involvement of locally-released neutrophins which promote, via their specific receptors, cancer cell proliferation and pro-survival signalling pathways. Thus, during CRC development cancer cells and neurons of the ENS release many neurotransmitters/neuropeptides which affect key cellular signalling pathways promoting cancer cell proliferation and pro-survival phenotype. The multiple interactions between ENS neurons, cancer cells and other cell types present in the colon wall increase cancer cell invasiveness and have a negative impact on the course of CRC.

## 1. Introduction: General Features of Cancer Invasion

Colorectal cancer (CRC) represents an invasive cancerous process which starts within the large intestine and is caused by the constant proliferation of epithelial cells which acquire the neoplastic phenotype. During this process, the epithelial cells lose the proper nature (a compact and regular arrangement and shape, cellular polarity, intercellular junctions and adherence to the basal lamina) and achieve the characteristic features of mesenchymal cells (oval or fusiform shape, lack of polarisation and cytoskeleton reorganisation). Such morphological and functional cell transformation is referred to as the epithelial-to-mesenchymal transition (EMT).

Neoplastic tumour formation within the large intestine wall is related to the replacement of normal tissues by cancer cell mass. In microscopic observations, two types of tumour invasive fronts have been classified and described, the first type is a “pushing tumour border” in which a well-demarcated tumour border extends and pushes normal tissues with a desmoplastic stromal reaction [1]. The second type is an “infiltrative tumour border configuration” in which irregular cancer cords penetrate into the lining tissue and a clear boundary between infiltrating and normal tissues is not visible. Frequently, such projections of cancer tissue at the invasive front are led by singular cancer cells or small clusters of cancer cells which are defined as “tumour buds” [1]. The different types of cancer invasion are related to different molecular backgrounds of cancer cells, which results in different courses of the illness. Infiltrative tumour border configuration is mostly associated with vascular invasion, lymph node and distant metastases and is correlated with poor patient prognosis (shorter overall survival, OS and disease-free survival, DFS) [1,2]. Subsequently, the cancer invasion destroys layers of the colon wall and forms a solid tumour which causes clinical symptoms correlated with the dysfunction of this part of the intestine.

Cancer cells may produce and secrete proteolytic enzymes (proteinases) which digest the epithelial basement membrane (BM) and the extracellular matrix (ECM) of the underlying connective tissue. BM destruction defines the invasive potential of the cancer. This activity is possible because colon cancer cells produce matrix metalloproteinase 2 (MMP-2) and matrix metalloproteinase 9 (MMP-9), gelatinases which dissolve type IV collagen, the essential component of BM [3,4]. The MMP-9 protein expression in CRC tissue is higher than in adenomas or the normal colon mucosa and its expression in a tumour tissue significantly correlates with clinical data: cancer grade, higher risk of disease recurrence and shorter survival of CRC patients [5,6]. The findings of MMP-9 presence not only in the primary tumour but also in the tumour budding strongly supports the hypothesis that proteolytic activity helps to create an “infiltrative tumour border configuration” [7].

A cancer invasion which destroys and disintegrates the stromal tissue interacts with cells located in this area. Stromal cells, including fibroblasts, mesenchymal stem cells, mast cells and transient cells such as blood-derived leukocytes (mainly macrophages, neutrophils and lymphocytes) are found in this location and, together with cancer cells, constitute a specific cancer milieu known as the tumour microenvironment (TM). Colon cancer cells interact with these residential and transient cells through different types of cell communication via soluble ligands or extracellular vesicles as well as cell–matrix interactions. This multi-stage process of mutual communication eventually promotes cancer growth and invasion, induces EMT, initiates angiogenesis and develops significant tolerance of the immune system to tumour antigens. In the course of cancer invasion, infiltrative tumour degrades gradually the innervation of the intestine and causes atrophy of the enteric nervous system components.

## 2. Morphology and Neurotransmission within the Enteric Nervous System

In normal conditions, the numerous nervous ganglia are regularly displaced between the particular layers of the large intestine wall. They form the enteric nervous system (ENS) which innervates the particular layers of the gastrointestinal (GI) tube wall. The spatially arranged, regularly distributed ganglia of the ENS are interconnected by nervous fibres and regulate the proper physiological functions of the intestine.

**Figure 1:**
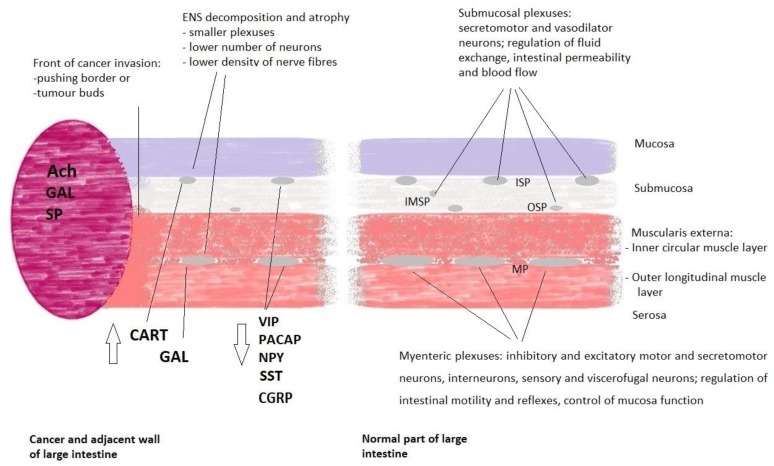
Major structural components and function of the enteric nervous system in human large intestine close and distant from the colorectal cancer mass. On the left side, cancer tumour and adjacent colon walls with white arrows showing the direction of changes in the expression of neuropeptides as compared to the normal intestine at the resection margin. On the right side, location of submucosal and myenteric plexuses in the normal large intestine. For clarity efferent and afferent nerve fibres are not shown. Diagram illustrates major cancer-related alterations of the ENS and major functions of the ENS plexuses as detailed in the main text. Abbreviations: Ach—acetylcholine, CART—cocaine and amphetamine-regulated transcript peptide, CGRP—calcitonin gene-related peptide, ENS—enteric nervous system, GAL—galanin, IMSP—intermediate submucosal plexus, ISP—inner submucosal plexus, MP—myenteric plexus, OSP—outer submucosal plexus, NPY—neuropeptide Y, PACAP—pituitary adenylate cyclase-activating polypeptide, SP—substance P, SST—somatostatin, VIP—vasoactive intestinal peptide.

Morphologically, the human ENS consists of plexuses and nerve fibres located in the submucosa and muscularis externa of the intestinal wall. The submucosa contains a complex of three small ganglia: the inner submucosal plexus (ISP; Meissner’s plexus), adjacent to the muscularis mucosae, the intermediate submucosal plexus (IMSP) and the outer submucosal plexus (OSP) adjacent to the muscularis externa. Furthermore, significantly larger clusters of neurons and glial cells, which form the myenteric plexus (MP; Auerbach’s plexus), are located between two layers of the muscularis externa [8] (Figure 1).

In humans, the number of ENS neurons is about 500 million, many more than in the other parts of the peripheral autonomic nervous system [9]. The longitudinal and circular layers of the muscularis externa are innervated by nerve fibres of sympathetic extrinsic ganglia and nerve fibres of Auerbach′s plexus motoneurons and such organisation regulates proper intestinal motility. Moreover, the peristaltic reflexes of the intestine are coordinated by an abundant network of the myenteric plexuses descending and ascending nerve fibres [8]. The intestinal mucosa is innervated by fibres of secretomotor neurons originating from submucosal plexuses. Secretomotor neurons regulate fluid exchange, intestinal permeability and coordinate proper intestinal reflexes. The lumen of submucosal blood vessels is regulated by axons of the vasomotor neurons (dilatory function) of submucosal plexuses and/or nitrergic (neuronal nitric oxide synthase, i.e., nNOS) neurons and axons of sympathetic neurons of extrinsic ganglia (constrictory function) [9,10]. Moreover, all the layers of the intestine and blood vessels are innervated by the sensory neurons (with mechano-, thermo- and chemoreceptors) of submucosal and myenteric plexuses as well as dorsal root ganglia. All components of the ENS are interconnected by numerous interneurons which functionally integrate them into one system which functions are based on physiological reflexes [11,12].

Acetylcholine (Ach) is the main neurotransmitter in the ENS neurons which stimulates secretory activity of the intestine and contraction of smooth muscle in the wall of the GI tube. It is present in almost all types of neurons: excitatory muscle motor neurons, secretomotor neurons, sensory neurons and some interneurons. The opposite action has vasoactive intestinal polypeptide (VIP), a neurotransmitter which causes relaxation of smooth muscles, especially in the wall of blood vessels (vasodilatory function) [11]. The actions of these two basic neurotransmitters are modified by the activity of about 30 other neurotransmitters (neuromodulators) which are present in the neurons of the ENS. Most neurons show the simultaneous expression of 2–3 neurotransmitters/neuromodulators, but some groups of neurons express larger numbers of neuropeptides. Generally, co-activity and modulatory action of many neurotransmitters are known as chemical coding ensuring flexibility of intestinal functions [8,9,10].

## 3. Cancer and the ENS Decomposition

The neuronal responses within the ENS are based on composed reflexes and multimodal transmissions, and they are modified under pathological conditions. It is known that parasite infections [13] and inflammatory bowel disease (IBD) cause prominent changes in the ENS including altered expression of neurotransmitters/neuropeptides within neurons [14]. In such clinical conditions, many ENS neuropeptides may act as proinflammatory or anti-inflammatory factors affecting cytokine secretion, maintenance of intestinal permeability and promotion of neuron survival by neuropeptides [15].

Moreover, it was found that ENS plasticity occurs also during colorectal cancer development [16]. A wide range of changes in the submucosal and myenteric plexuses components located close to the front of the CRC invasion was observed. These changes involve a decreased number of neurons and/or nerve fibres containing several neuropeptides: VIP, pituitary adenylate cyclase-activating polypeptide (PACAP), neuropeptide Y (NPY), somatostatin (SST/SOM), substance P (SP) and calcitonin gene-related peptide (CGRP) [17,18]. The alterations of these neurotransmitters in colorectal cancer cell lines, tumour tissue, blood and experimental animal models are summarised in Table 1.

Table 1 Function and localisation of the ENS neurotransmitters in the normal human large intestine and colorectal cancer cell lines and tumour tissue. ACh—acetylcholine, CASP8—caspase 8, CGRP—calcitonin gene-related peptide, CRC—colorectal cancer, EGF—epithelial growth factor, ERK—extracellular signal-regulated kinase, ENS—enteric nervous system, FLIP—FLICE-inhibitory protein, GAL—galanin, GRP—gastrin related peptide, IHC-immunohistochemistry, 3mAChR—muscarinic receptor type 3, MAPK—mitogen-activated protein kinases, MMP7—matrix metalloproteinase 7, NK1Rs—neurokinin-1 receptors, NPY—neuropeptide Y, PACAP—pituitary adenylate cyclase-activating polypeptide, PI3Κ—phosphoinositide 3-kinase, SP—substance P, SST/SOM—somatostatin, VIP—vasoactive intestinal peptide.

Interestingly, we found that the number of neurons containing galanin (GAL) within myenteric plexuses located in the vicinity of the infiltrating cancer was higher compared to the MPs in a region distally located from the tumour margin [65]. Moreover, the relative number of cocaine and amphetamine-regulated transcript peptide (CART)-immunoreactive neurons increased by three-fold in OSP, and ISP [66] located in cancer invasion boundary.

There are distinct differences in the histological structure and location of two basic types of the ENS plexuses within the intestinal wall: small submucosal plexuses of oval or irregular shape are present in a loose structure of the submucosal connective tissue, while large and elongated myenteric ganglia are regularly deployed in a very narrow space between the inner circular and the outer longitudinal muscle layers of the muscular externa. The cancer invasion gradually destroys the normal morphology of the colon wall including the entire innervation located in the invasive area. Furthermore, our microscopic observations indicated that cancer infiltration causes displacement and subsequent atrophy of the submucosal and myenteric plexuses in the region adjacent to the tumour boundary [67]. This observation was confirmed by morphometric analyses which demonstrated that MPs located in close proximity to the cancer invasion were significantly smaller and had a lower number of neurons per plexus than the MPs located farther from the tumour [60]. Moreover, electron microscopy analysis demonstrated that in the MPs located close to the cancer invasion, expansion of extracellular matrix and a sporadic presence of apoptotic neurons were observed [68]. Ciurea et al. observed a decrease of the relative area for both Auerbach and Meissner plexuses with the increase of the tumour grading as well as an increase of the relative area of other nervous elements not in the Meissner plexus or in the Auerbach plexus with the tumour grading [69].

To date, the mechanism which causes the atrophy of myenteric ganglia in the colon wall of patients with CRC has not been clearly elucidated. The stroma reaction, with a tumour mass-related increased local tissue pressure with simultaneous hypoxia, could be considered as a potential factor of such atrophy. It could be also promoted by cancer cells located close to the ganglia via paracrine signalling. Such possibility seems to be supported by the results of our study of the colon samples of patients with CRC in which we observed an increased number of neurons with caspase 8 immunoreactivity (CASP8-Ir) within the myenteric ganglia in the vicinity of tumour tissue [70]. CASP8 is triggered by binding of the extracellular ligands to a cell membrane receptor which activates the extrinsic pathway of apoptosis. In the same plexuses, we observed a significantly lower number of neurons with caspase 3 (CASP3) immunoreactivity than those with CASP8-Ir (26% vs. 46%), which may suggest the extrinsic apoptotic pathway had been silenced at the terminal, executive phase of apoptosis. The fact that almost all neurons expressing CASP3 also demonstrated the presence of GAL indicates a possible role for this neuropeptide in the observed phenomenon [70]. Thus, atrophy of myenteric plexuses may be caused by stromal reaction and tissue decomposition, which is observed at the front of cancer invasion as well as by molecules originating from cancer cells which are released into the tumour environment as secretome and/or extracellular vesicles.

## 4. Cancer Cell Proliferation and Apoptosis Inhibition Related to the ENS Neurotransmitters/Neuromodulators

The process of colorectal cancer development is a multi-stage process. Neoplastic changes occur at the cellular level (proliferation, lack of cell differentiation and lack of apoptosis) and at the tissue level (the front of the invasion and angiogenesis) during the local phase of cancer development. Similarly to other neoplasms, CRC is a genetic disorder that is caused by the sequential imposition of several subsequent mutations. The key initial mutation in the adenomatous polyposis coli (*APC*) gene mutation, which is followed by sequences of *KRAS, p53* and delated in colorectal cancer *(DCC)* gene mutations.

The initial point is the *APC* suppressor gene mutation, found in many patients suffering from colorectal cancer, including patients with the hereditary familial adenomatous polyposis (FAP) disorder. APC protein binds to β-catenin within the cytoplasm and causes its degradation within proteasomes. β-catenin is a transcription factor which, when up-regulated (in the case of lack of degradation), induces constant proliferation of intestinal epithelial cells. The expression of the β-catenin protein results from the activation of the Wnt/β-catenin pathway via a frizzled receptor (a G protein coupled-receptor type). Wnt signalling is essential for the differentiation of intestinal epithelial cells and their migration from base to apical regions of epithelial crypts. Disruption of the Wnt/β-catenin pathway leads to excessive epithelial cell proliferation and inhibition of differentiation, which results in the development of intestinal polyps [71].

A possible relationship between Wnt/β-catenin signalling and colon cancer cell proliferation may result from acetylcholine action via muscarinic receptors (mAChRs) [72]. Muscarinic AChRs belong to G protein-coupled receptors (GPCRs), similarly to neuropeptide receptors. There are five subtypes of mAChRs recognised, three of them are present in the alimentary tract: subtypes 1 and 3 are present in the GI tract epithelium (they activate phospholipase C signalling) and subtype 2 is present in smooth muscles (it inhibits adenyl cyclase which results in decreased cAMP concentration) [73,74]. The connection between acetylcholine and Wnt signalling within epithelial cells is associated with subtype 3mAChR and conservative transcription factor (ELK), which cause Wnt/β-catenin pathway activation in autocrine and paracrine manners [75].

In the tumour microenvironment, acetylcholine could originate from two sources-released by cholinergic neurons of the ENS and from non-neuronal tissues. It was shown that colon cancer cells can produce acetylcholine and demonstrate the over-expression of subtype 3mAChR. These two factors (ligand presence and/or receptor over-expression) have a significant impact on cancer cell proliferation [22]. Furthermore, the activation of mAChR causes MMP-7 release, which cleaves pro-EGF into an active ligand form [76]. Subsequently, epithelial growth factor (EGF) reacts with the EGF receptor (tyrosine kinase receptor, RTK) and activates two key mitogenic pathways PI3K/PKC/NF-kB and Ras/Raf/MEK/MAPK/ERK what results in increased cell proliferation. Moreover, this may affect cancer cell survival because phosphoinositide 3-kinase/serine/threonine kinase (PI3K/Akt) signalling could attenuate apoptosis via up-regulation of pro-survival genes and down-regulation of pro-apoptotic proteins [77].

The next key alternation in the canonical CRC development pathway are mutations in the *KRAS* gene which occur at the early stage of CRC carcinogenesis. The KRAS protein belongs to the mitogen-activated protein kinase (RAS) family and activates the mitogen-activated protein kinases (MAPK) pathway. As a result of *KRAS* mutation, the protein is present only in the activated form (GTP-coupled) and, thus, continuously activates the MAPK cascade, independently of extracellular factors [78]. MAPK pathway up-regulation may also occur at the specific membrane receptor tyrosine kinase level. Continuous stimulation of this receptor may be caused by an increased presence of growth factors, e.g., transforming growth factor beta (TGF-β) secreted by tumour-associated macrophages. It was shown that the addition of a neuropeptide VIP to the cultures of the CRC cell line caused cell proliferation via the activation of the MAPK pathway [30]. Furthermore, there is a link between the activation of the G protein receptor (specific for neurotransmitters/neuromodulators) and the MAPK pathway upregulation. This means that the MAPK mitogenic pathway up-regulation is not only related to the presence of the mutated KRAS active form but may also result from RKT stimulation by growth factors or neurotransmitter–G protein receptor interaction.

Studies of experimental nerve injury (axotomy) and acute inflammation of the large intestine in pigs demonstrated a higher galanin presence within the large intestine wall and its ganglia [79,80] which strongly suggests a neurotrophic role for this neuropeptide. It is known that GAL binding by its GALR1 and GALR2 receptors initiates a cascade of intracellular signalling pathways, resulting in cell proliferation [81]. Activation of GAL receptors via protein kinase C (PKC) activates the Ras/MAPK/ERK pathway by increasing intracellular calcium ion concentration. Furthermore, activation of GAL receptors causes the initiation of the PI3K/Akt-dependent pathway which results in cell survival [81]. In this way, GAL could not only be a neuroprotective factor, but it also can play an important role in cancer progression. The possible role of GAL in CRC pathogenesis is supported by a report which documented increased *GAL* gene and GAL protein expression in the CRC tissue [58,60]. Such gene and protein up-regulation within CRC tissue may suggest that GAL is produced and secreted by cancer cells and acts as a mitogen within cancer tissue in an autocrine way.

In a human study, *GAL* expression at the mRNA level was up-regulated in the tumour tissue and correlated with poor prognosis and tumour recurrence in CRC patients in stage II of the disease [61]. Moreover, siRNA-mediated silencing of the *GAL* gene decreased proliferation and invasive potential of CRC cell lines [59]. An *in vitro* study showed that GAL and GALR1 silencing induced apoptosis in drug-sensitive and drug-resistant six independent CRC cell lines. GALR1 silencing caused down-regulation of an endogenous CASP8 inhibitor, FLIP_L_, and consequently, the activation of the external apoptosis pathway and increased apoptosis of cancer cells. Thus, FLIP_L_ may be a key downstream effector of GalR1/galanin-mediated anti-apoptotic signalling [59]. Other studies of GAL and its receptors also suggest the role of GAL as a cancer-promoting factor in non-CRC neoplasms. For example, in cultures of small-cell lung cancer cell lines, GAL was found to act as a cancer growth factor by stimulation of the MAPK mitogenic pathway [82]. Studies of head and neck squamous cancer cell lines demonstrated increased proliferation and survival of these cells as a result of GALR2 over-expression and activation of MAPK/ERK and PI3K/Akt-dependent signalling [83].

Blood vessels of mucosa and submucosa of the large intestine are innervated by sensory nerves of intrinsic primary afferent neurons (IPAN) which are located in the submucosal and myenteric plexuses. These are cholinergic neurons with SP-neurochemical coding. Moreover, extrinsic sensory nerve fibres of the dorsal root ganglia display both SP and CGRP presence. Reflexes of the large intestine caused by the distension of its wall are highly autonomic and support IPAN and SP transmission/modulation roles in such a function [8]. SP is not only present in sensory neurons of the ENS but it is also located in CRC cells. It was found that SP high expression in CRC tissue is correlated with cancer metastasis and shorter patients’ survival [48]. Substance P reacts with cells via neurokinin-1 receptors (NK1Rs) which belong to GPCRs. NK1Rs are widely expressed in various cells which are present in large intestine tissues: neurons, epithelial cells, smooth muscle cells, fibroblasts and immune cells. NK1R activation by SP is a complex process involving plasma membrane vesicles internalisation and recycling (receptor turnover), resulting via second messengers (PI3K and cAMP) in MAPK pathway activation. SP also induces cell proliferation of lymphocytes, macrophages and endothelial cells [84]. NK1Rs are also present and overexpressed within colon cancer cells [46]. Such up-regulation of these receptors may trigger the proliferation of colon cancer cells involving the EGFR kinase domain and MAPK pathway signalling [85].

## 5. Cancer Perineural Invasion

It is a well-known morphological observation that cancer invasion can spread along nerve fibres in many neoplasms, especially prostate cancer and pancreas cancer. In the CRC, such type of perineural invasion (PNI) is observed in about 20% of patients and is concerned as a feature of cancer invasiveness potential [86]. Perineural invasion is correlated with other histological features of cancer development such as tumour growth pattern, tumour budding presence, tumour grade (T feature) and regional lymph metastasis (N feature) and lymphatic and blood vessel invasion [87]. Moreover, PNI may affect the clinical course of CRC since a correlation with its presence and shorter patient survival was observed and found to be an independent prognostic factor of patient poor prognosis [87]. Furthermore, it could be used as a factor which can result in clinical decisions related to the use of chemotherapy in clinical stage II CRC patients [88]. A recent *in vitro* study by Duchalais et al. [89] carried out on the co-cultures tumour epithelial cells (TEC) from human primary colon adenocarcinomas and cell lines with primary cultures of ENS and cultures of human ENS plexus revealed that TEC adhered preferentially and with stronger adhesion forces to the ENS structures than to mesenchymal cells and that the adhesion to ENS involved direct interactions with enteric neurons. Moreover, these authors showed that blocking N-cadherin and L1CAM decreased TEC migration along ENS structures, and therefore, concluded, that the enteric neuronal network guides tumour cell migration [89].

The second factor supporting the correlation of cancer development and innervation of the large intestine may be derived from the studies of Chagas disease. In the course of *Trypanosoma crusi* infection, there is severe ENS damage and degeneration which causes widening of the large intestine (megacolon) and pathological constipation. The slow passage of faeces was suggested to be a risk factor in colon carcinogenesis since it enables prolonged exposition of the intestinal epithelium to carcinogens present in stool. However, it was clearly demonstrated that the risk of CRC is significantly lower in the course of Chagas disease than in the normal population [90]. For example, in 894 chagasic patients who were operated on for megacolon resection in one San Paulo (Brazil) hospital from 1952 until 2001 any type of colonic neoplasm was found, despite the presence of other CRC risk factors such as mucosal ulcers, hyperplasia and chronic inflammation [91]. Interestingly, atrophy of myenteric plexuses and gliosis characterise the morphology of colon wall in Chagas disease [90,91,92], and the ensuing loss of innervation within colon wall was implicated as the factor which prevents cancer development in patients with Chagas disease [92].

In analysing the PNI phenomena in CRC invasion, it could be assumed that cancer cells proliferate and invade colon tissue by actively migrating along axons in a mechanism called neural tracking. The ability of cancer cells to track along nerves is facilitated by multiple growth factors and cytokines secreted by various cell types in the perineural niche [93]. Moreover, the observations of nerve fibre atrophy close to cancer infiltration [67] may also suggest that the degenerative changes of fibres which are caused by external factors may result in neurotrophic factor secretion. It is possible that these factors interact with cancer cell invasions and stimulate those cells to proliferate which predominate in the perineural area due to a higher concentration of neurotrophic factors along nerve fibres.

Within the ENS of the human large intestine, essential neurotrophic factors such as nerve growth factor (NGF), brain-derived neurotrophic factor (BDNF) and neurotrophin 3 (NT-3) are normally present [94]. They not only affect neuron migration and axon guidance during the development of gut innervation in the embryonal period but play an active role also in such diseases as IBD, irritable bowel syndrome (IBS), *Trichinella spiralis* infection, bowel obstruction and diabetes-induced enteric neuropathy [94]. In the colon wall, neutrophins eutrophins are secreted not only by the ENS neurons but also other cell types such as enteric glial cells, epithelial cells, lamina propria cells and cells of the immune system [94].

Neurotrophins control differentiation, plasticity and neuron survival via two different types of receptors: the p75 neurotrophin receptor which belongs to the tumour necrosis factor receptor family and three tropomyosin receptor kinases (Trk) which belong to the RTKs family [95]. P75 is the receptor for all four (including NT-4) neurotrophins whereas NGF binds TrkA, BDNF and NT-4 bind TrkB and NT-3 mainly binds TrkC [95]. These Trk receptors activate three key signalling pathways related to cell survival: MAPK/ERK, PI3K/Akt and phospholipase C gamma pathways [95,96]. It was found that both BDNF [97] and TrkB receptors [97,98] are overexpressed in colon cancer tissue, especially in advanced clinical stages of disease [97]. BDNF and TrkB agonists increased the proliferation of CRC cell lines and showed anti-apoptotic activity [97,98]. Moreover, the up-regulation of the TrkB receptor in colon cancer cells was correlated with higher lymphatic vessel density and metastasis [98]. Thus, BDNF/TrkB signalling inhibition could be a new therapeutic target in the treatment of CRC [99,100] and other digestive tract cancers [101].

**Figure 2:**
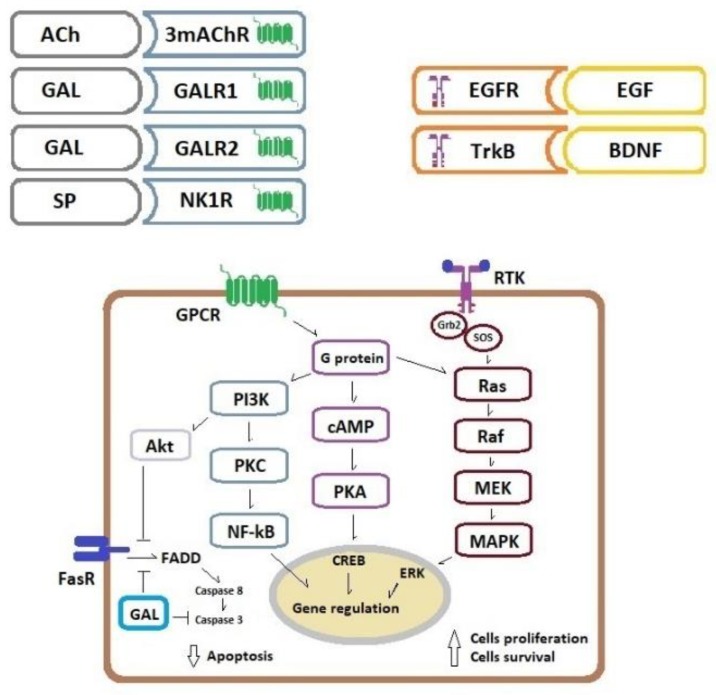
Schematic presentation of key pathways activated in a colorectal cancer cell by three groups of cell membrane receptors. Binding of Ach, GAL and SP to GPCRs triggers multiple transduction pathways which promote cell proliferation and survival, binding of EGF and neutrophins to the RTKs promotes cell proliferation whereas binding of FasR ligands activates extrinsic pathway of apoptosis. A more detailed description is in the main text. Abbreviations: Ach—acetylcholine, AKT—serine/threonine kinase, BDNF—brain-derived neurotrophic factor, CREB—cAMP response element-binding protein, EGF—epidermal growth factor, EGFR—EGF receptor, ERK—extracellular signal-regulated kinase, FADD—Fas-associated protein with death domain, FasR—Fas receptor, GAL—galanin, GALR1—galanin receptor 1, GALR2—galanin receptor 2, GPCR— G protein–coupled receptor, Grb2—Growth factor receptor-bound protein 2, MAPK—mitogen-activated protein kinases, MEK—MAPK/ERK kinase, 3mAChR—muscarinic receptor, NF-kB—nuclear factor-kappa B, NK1R—neurokinin-1 receptor, PI3Κ—phosphoinositide 3-kinase, PKA—protein kinase A, PKC—protein kinase C, RAF—mitogen-activated protein kinase, RAS—mitogen-activated protein kinase, RTK—receptor tyrosine kinase, SOS—Son of sevenless, SP—substance P, TrkB—tropomyosin-related kinase receptor B.

The basic intracellular pathways activated in the colorectal cancer cells by acetylcholine, neuropeptides and growth factors are presented in Figure 2.

## 6. Conclusions

Colorectal cancer infiltrates the intestinal tissue and evokes reactions of stromal cells present in the tumour’s microenvironment. Invasive tumour mass causes atrophy of the components of the enteric nervous system located in the adjacent area. The neuroprotective reaction of neurons and glial cells results in the secretion of neuromediators and neurotrophic factors which affect cancer cells by stimulating their proliferation and perineural invasion. Such positive feedback significantly affects the invasiveness of cancer, which results in a worse prognosis and course of the disease.

## Figures and Tables

**Table 1:** Function and localisation of the ENS neurotransmitters in the normal human large intestine and CRC cell lines and tumour tissue.

ACh	Normal function in the large intestine
	Stimulates contraction of muscularis externa smooth muscles; increase fluid secretion and blood flow in submucosa vessels, regulates proper intestinal peristalsis and reflexes [10,19,20,21].
	Localization in the ENS: excitatory muscle motor neurons, secretomotor neurons, viscerofugal mechanosensory neurons and interneurons, vasodilator neurons [8,9].
	Possible involvement in colorectal cancer development: ACh stimulates cancer cell proliferation in an autocrine and paracrine way.
	*In vitro* studies on CRC cell lines
	- H508 cells; nonselective muscarinic receptor antagonists, selective 3mAChR antagonist, and choline transport inhibitors attenuate cancer cells proliferation; acetylcholinesterase inhibitors induce cell proliferation [22].
	- H508, WiDr, and Caco-2 cells and conditioned-cell culture media; choline acetyltransferase induces cell proliferation [22];
	- H508 and HT29 cells; ACh induces cell migration mediated by MMP-7, which cleaves pro-EGF into EGF, EGFR activation and MAPK/ERK and PI3K signaling [23].
	CRC tumour tissue and *in vivo* studies
	- 3mAChR-deficient mice, chemically-induced CRC model; a lower number of adenoma and adenocarcinoma and lower tumour size [24].
	- in human CRC 3mAChR over-expression (IHC) was found in 70% of adenomas vs adjacent normal epithelium; in CRC tumour cells both intense apical and cytoplasmic immunoreactivity (Ir) vs only apical, weak Ir in normal colon epithelium; no alterations of 3mAChR-Ir in lymph node and liver metastases [25];
VIP	Normal function in the large intestine
	As potent vasodilator increases blood flow in submucosal vessels [19,26]; is the primary pro-secretory neurotransmitter in the large intestine [19,26] by increasing colon electrogenic Cl^–^ and HCO3^–^ secretion [27]; affects intestinal barrier function [26]; has anti-inflammatory effects [28]; relaxes of smooth muscles of muscularis externa [9,10].
	Localization in the ENS: inhibitory motor neurons, secretomotor neurons, viscerofugal neurons, part of interneurons and vasodilator neurons [8,9,21].
	VIP receptor (VPAC1) mRNA expression in the human intestine, was highest in the sigmoid colon, followed by ileum and jejunum; in human colon VPAC1 showed predominantly apical localization in the intestinal epithelium [29].
	Possible involvement in colorectal cancer development: VIP stimulates cancer cell proliferation, but has anti-metastatic activity and attenuates angiogenesis.
	*In vitro* studies on CRC cell lines
	- HT29 cells; VIP stimulates cells proliferation and induced a time- and concentration-dependent activation of cAMP-Rap1/Ras-B-Raf-ERK signalling pathway [30].
	- HCT-15 cells; VIP antagonists, neurotensin(6-11)VIP(7-28) - inhibitor of cancer cells growth [31].
	- 26-L5 cells; VIP attenuate cancer cells motility and invasiveness potential [32].
	Tumour tissue and *in vivo* studies
	- Human CRC tumours: VPAC1 receptors overexpression (IHC) is associated with poor differentiation in CRC; VPAC1 receptors prevail in blood vessels surrounding tumour CRC and are numerous in tumour-associated macrophages [33];
	- Mice, experimental CRC metastasis model; VIP inhibits liver metastasis in a dose-dependent manner and attenuates angiogenesis through suppression of endothelial cells [34,35];
	- Mice, chemically induced CRC; VIP increases the incidence of colonic tumours [36];
	- Rat, chemically induced CRC, administration of VIP hybrid antagonist neurotensin(6-11)VIP(7-28) for 10 weeks reduced tumour volume, staging, lymphocyte infiltration and the number of dysplastic crypts [31].
PACAP	Normal function in large intestine
	Increased intestinal secretion [9,19,37] and relaxation of smooth muscle of muscularis externa [38].
	Localization in the ENS: inhibitory motor neurons and part of interneurons; PACAP is frequently colocalized with the sensory neuropeptide CGRP and also with VIP [8,9].
	Possible involvement in colorectal cancer development: PACAP stimulates cancer cell proliferation and induces cell survival.
	*In vitro* studies on CRC cell lines
	- HCT8 cells; PACAP-38 elevates via PAC1 receptor both intracellular cAMP levels and cytosolic Ca(2+) concentration, increases the number of cancer cells, attenuates apoptotic signaling via Fas-R/Fas-L down-regulation [39].
	Tumour tissue and *in vivo* studies
	PACAP38- and PACAP27-like immunoreactivity was lower in tissue samples of of CRC compared with normal colon tissue [40].
	PACAP-deficient mice, rapid development of CRC tumours in chemically induced CRC experimental model [41].
NPY	Normal function in large intestine
	Increased intestinal secretion, regulation of blood flow in the submucosa, relaxation of muscularis externa [19,21,42], proinflammatory effects [28]
	Localization in the ENS: inhibitory motor neurons, secretomotor neurons and innervation of submucosal blood vessels [8,9].
	Possible involvement in colorectal cancer development: reduces cancer cells invasiveness.
	*In vitro* studies on CRC cell lines
	- 26-L5 cells; NPY suppresses cancer cell motility, inhibits cell migration, reduces invasive potential of tumour cells in a concentration-dependent manner [32].
	Studies on CRC tumour and blood samples, CRC biomarker, *in vivo* studies
	- Hypermethylated NPY gene, is a marker to screen patients with high risk of colorectal cancer [43], and hypermethylated NPY circulating tumour DNA (MetctDNA), is the useful marker of cancer development [44].
	- NPY promotes inflammation-induced tumorigenesis (DSS-mouse model) by increasing epithelial cell proliferation and downregulating apoptosis [45]
CGRP	Normal function in large intestine
	Intestinal sensation and modulation of intestinal secretion [19,21]
	Localization in the ENS: sensory neurons and secretomotor neurons and innervation of submucosal blood vessels [8,9].
	Possible involvement in colorectal cancer development: reduces invasive potential of cancer cells.
	*In vitro* studies on CRC cell lines
	- 26-L5 cells; CGRP suppresses cancer cell motility, inhibits cell migration, reduces invasive potential of tumour cells in a concentration-dependent manner [26]
SP	Normal function in large intestine
	Modulation of intestinal secretion and intestinal sensation [19], contraction of muscularis externa [21], proinflammatory effects [28].
	Localization in the ENS: excitatory muscle motor neurons, sensory neurons and part of interneurons [9].
	Possible involvement in colorectal cancer development: SP increases cancer cell proliferation.
	*In vitro* studies on CRC cell lines
	- 23132/87 and SW-403 cells; L-733,060, neurokinin-1 (NK-1) receptor antagonist - inhibit the growth of CRC cells in a dose-dependent manner via activation apoptosis [46].
	- LiM6 and DLD1 cells; Upregulation of SP-NK1R – induce CRC progression, neurokinin-1 receptor (NK1R) antagonist - inhibit colon cancer cell lines growth via inhibition Wnt signaling [47].
	Tumour tissue and *in vivo* studies
	- CRC tissue sample; SP and NK1R levels is upregulated in CRC, high expression is associated with lymph node metastasis and poor prognosis [48].
SST (SOM)	Normal function in large intestine
	Inhibition of intestinal secretion and intestinal motility, blood flow regulation in the submucosa [19,49]; anti-inflammatory effects [28]
	Localization in the ENS: secretomotor neurons, part of interneurons, submucosal blood vessels [9,21].
	Possible involvement in colorectal cancer development: STT inhibits growth of colon cancer cells.
	*In vitro* studies on CRC cell lines
	- Caco-2, HT-29 and HCT116 cells; STT inhibit cell proliferation, decreases COX-2 expression and function in CRC cells via activation of sst(3) or sst(5) receptors [50].
	- SW480 cells; Octreotide, (STT analog), downregulate Wnt target genes cyclinD1 and c-Myc [51], inhibit Wnt/beta-catenin signaling pathway - arrest the cell cycle and induce apoptosis [52].
	- HT-29, HCT-15, and HCT-116 cells; AN-162 (SST analog) increase the number of apoptotic cells [53].
	Studies on CRC tumour and blood samples
	- CRC tissue samples; SST receptor type 2 and 5 - negative correlation with CRC invasion and liver metastasis. Patients with longer survival [54].
	- CRC blood samples; methylated SST gene, at the preoperative time point - associated with patients shorter survival and predictor for cancer recurrence [55].
GAL	Normal function in large intestine
	Modulates intestinal secretion, contraction of muscularis mucosae, increases blood flow in the submucosa [19,56,57], anti-inflammatory effects [28].
	Localization in the ENS: secretomotor neurons and vasodilator neurons [8].
	Possible involvement in colorectal cancer development: GAL induces proliferation of cancer cells and improves cell survival
	*In vitro* studies on CRC cell lines
	- LOVO, HCT15, SW480 and SW620 cells: high levels of galanin expression [58];
	- HCT116, LS174T, RKO, HT29, SW620 and LoVo; GalR1/galanin silencing results in the downregulation of the endogenous caspase-8 inhibitor FLIP(L), which results in the induction of caspase-8-dependent apoptosis [59].
	Studies on CRC tumour and blood samples, CRC biomarker.
	- CRC tissue: increased GAL gene expression [58] and GAL levels [60] correlate with poor disease-free survival, poor prognosis and tumour recurrence of CRC patients [59,61]
	- CRC blood samples; 2.4 times higher GAL concentrations than in healthy control [60].
GRP	Normal function in large intestine
	Relaxation of muscularis externa [19].
	Localization in the ENS neurons: inhibitory motor neurons and part of interneurons [9,21]
	Possible involvement in colorectal cancer development: GRP attenuates CRC invasiveness.
	*In vitro* studies on CRC cell lines
	- Caco-2 and LS-174T cells; inhibiting RP/GRPR signalling increases invasiveness of cells [62].
	Tumour tissue
	- GRP/GRPR co-expression in all well-differentiated part of CRC tumours [63], GRP and GRPR expression (IHC) correlated with better patients survival [64].

Abbreviations are provided in the description to the table.
